# Supplementary material for: The causal role of circulating inflammatory markers in osteoporosis: a bidirectional Mendelian randomized study
Source: Front Immunol. 2024 Jul 18;15:1412298. doi: 10.3389/fimmu.2024.1412298 (PMC11291241; doi:10.3389/fimmu.2024.1412298)
Supplement: Supplementary file 1 [file DataSheet_1.zip › supplementary material/OSTEOPOROSIS-CIM/GCST90274834.tsv.gz_forest.pdf]

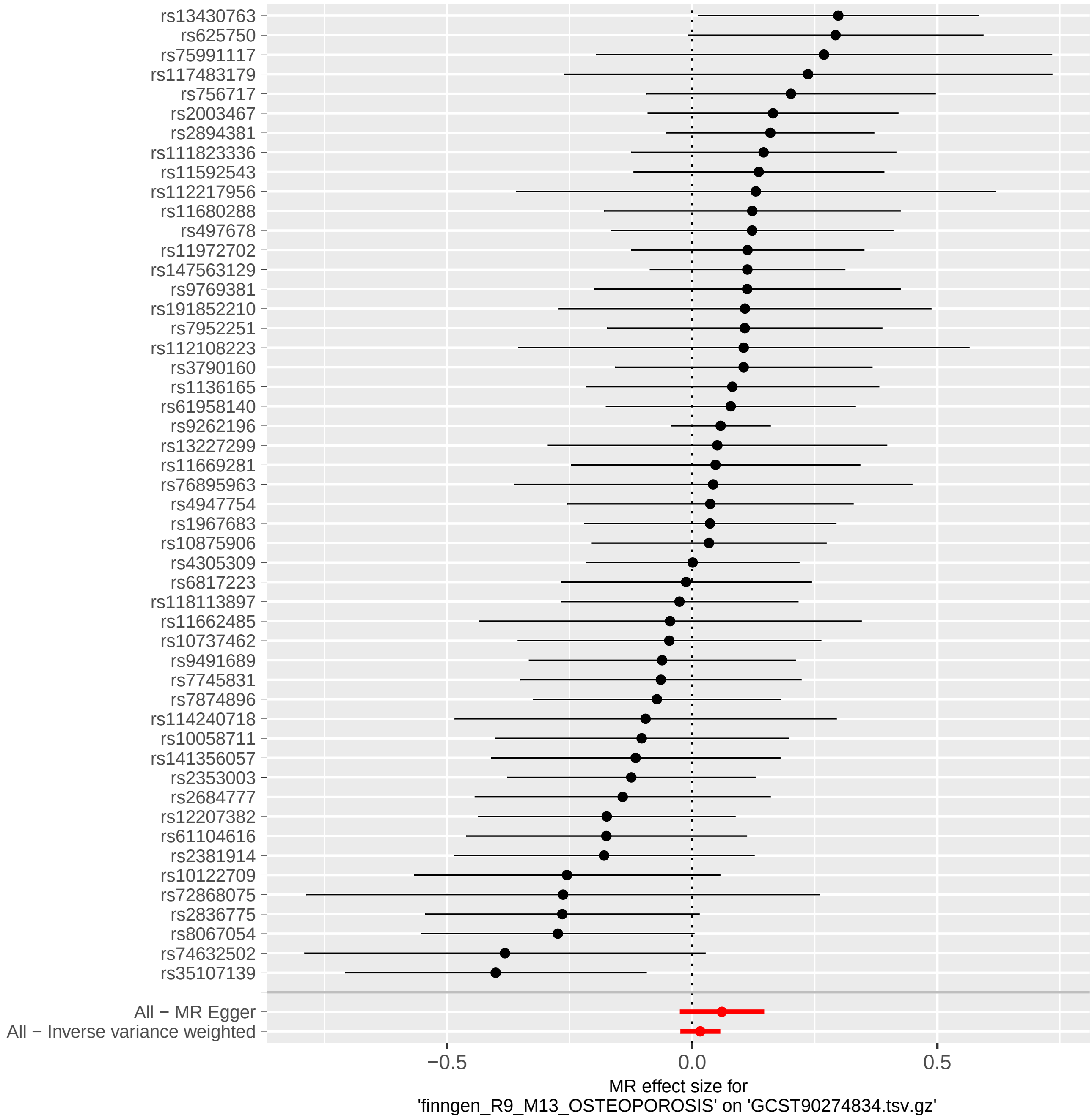
rs13430763
rs625750
rs75991117
rs117483179
rs756717
rs2003467
rs2894381
rs111823336
rs11592543
rs112217956
rs11680288
rs497678
rs11972702
rs147563129
rs9769381
rs191852210
rs7952251
rs112108223
rs3790160
rs1136165
rs61958140
rs9262196
rs13227299
rs11669281
rs76895963
rs4947754
rs1967683
rs10875906
rs4305309
rs6817223
rs118113897
rs11662485
rs10737462
rs9491689
rs7745831
rs7874896
rs114240718
rs10058711
rs141356057
rs2353003
rs2684777
rs12207382
rs61104616
rs2381914
rs10122709
rs72868075
rs2836775
rs8067054
rs74632502
rs35107139
All − MR Egger
All − Inverse variance weighted
−0.5
0.0
0.5
MR effect size for
'finngen_R9_M13_OSTEOPOROSIS' on 'GCST90274834.tsv.gz'
